# Racial/ethnic disparities on inflammation and response to methylprednisolone in severe COVID-19 pneumonia

**DOI:** 10.1186/s12879-022-07237-1

**Published:** 2022-03-14

**Authors:** Ronaldo C. Go, Themba Nyirenda, Maryam Bojarian, Davood K. Hosseini, Kevin Kim, Mehek Rahim, Elli G. Paleoudis, Anna C. Go, Zhiyong Han, Steven J. Sperber, Anjali Gupta

**Affiliations:** 1 Hackensack Meridian School of Medicine, Nutley, NJ USA; 2 Hackensack University Medical Center, Hackensack, NJ USA; 3 Gullas School of Medicine, Cebu, Philippines; 4 Department of Critical Care, Hackensack University Medical Center, Hackensack, NJ USA

**Keywords:** Racial/ethnic disparities, Comorbidities, Inflammation, Methylprednisolone, COVID-19

## Abstract

**Background:**

Racial/ethnic minorities are at higher risk for severe COVID-19. This may be related to social determinants that lead to chronic inflammatory states. The aims of the study were to determine if there are racial/ethnic disparities with inflammatory markers and association of methylprednisolone to in hospital survival.

**Methods:**

This was a secondary analysis of a retrospective cohort study of patients ≥ 18 years of age and admitted for severe COVID-19 pneumonia between March and June 2020 in 13 Hospitals in New Jersey, United States. Patients who received other formulation of corticosteroids were not included. Area under the receiver operating characteristics curves were performed to test for discriminatory ability of each inflammatory makers. Univariate and multivariate Cox regression assessed the association of variables to in hospital survival.

**Results:**

Propensity matched sample (n = 759) between no methylprednisolone (n = 380) and methylprednisolone (n = 379) had 338 Whites, 102 Blacks, 61 Asian/Indians, and 251 non-Black non-White Hispanics. Compared to CRP, area under receiving operating characteristic curve for d-dimer in Hispanics (0.742) was statistically different (DeLong Test P = 0.0041). Multivariate cox regression showed that different variables in Blacks [age ≥ 60 years (HR = 3.71, P = 0.0281), mechanical ventilation (HR = 5.07, P = 0.0281) and creatinine ≥ 1.5 mg/dL (HR = 3.61, P = 0.0007)], Whites [cancer (HR = 1.68, P = 0.0213), qSOFA score of 1 (HR = 1.81, P = 0.0213), qSOFA score of 2 (HR = 5.16, P < 0.0001), qSOFA score of 3 (HR = 11.81, P < 0.0001) and creatinine ≥ 1.5 mg/dL (HR = 2.16, P = 0.0006)], Hispanics [hypertension (HR = 2.52, P = 0.0007), cancer (HR = 2.99, P = 0.0244 and D-dimer ≥ 2 mcg/mL (HR = 2.22, P = 0.0077)], and Asian/Indians [ chronic kidney disease (HR = 6.36, P = 0.0031) and CRP > 20 mg/L (HR = 5.02, P = 0.0032)] were statistically significant for mortality. Low dose and high dose methylprednisolone were significantly associated with prolonged survival in Whites [low dose (HR = 0.37, P < 0.0001) and high dose (HR = 0.48, P < 0.0183)] and Asian/Indians [low dose (HR = 0.13, P = 0.0101) and high dose (HR = 0.15, P = 0.01)]. However, high dose was not associated with improved survival compared to low dose. Methylprednisolone was not associated with prolonged survival in Blacks and Hispanics.

**Conclusion:**

Racial/Ethnic disparities with inflammatory markers preclude the use of one marker as a predictor of survival. Methylprednisolone is associated with prolonged survival in Asian/Indians and Whites.

**Supplementary Information:**

The online version contains supplementary material available at 10.1186/s12879-022-07237-1.

## Introduction

As of July 2, 2021, there has been 182,319,261 coronavirus disease 2019 (COVID-19) cases and 3,954,324 deaths in worldwide [1], and the main cause of mortality is hyperinflammatory acute respiratory distress syndrome (ARDS). Advanced age, diabetes, cardiovascular disease, chronic lung disease, chronic kidney disease (CKD), and racial/ethnic minorities are among the factors that appear to increase the risk for severe COVID-19 [2–4]. Racial/ethnic minorities include non-black non-white Hispanics, Blacks, Native Americans, Native Hawaiians and other Pacific Islanders [5]. After age-related adjustments, mortality in Blacks, non-white non-black Hispanics, and Asians are higher compared to Whites in the United States, United Kingdom, and Brazil [1, 6–8].

One explanation is social determinants (multi-generational homes, essential workers, low socio-economic status, lack of access to quality health care) predispose these minorities to higher COVID-19 exposure, and behaviors (depression, anxiety, smoking, alcoholism, high sugar, salt and fatty diet) that lead to chronic inflammatory states. These might influence the severe clinical presentation [5]. Inflammatory markers, such as IL-6 (> 25 pg/mL), D-dimer (≥ 2.0 mcg/mL), CRP (≥ 20 mg/L) and/or ferritin ≥ 1400 μg/L, are believed directly correlate with mortality in COVID-19 [9–16]. There are racial/ethnic variability with inflammatory markers [17] but there has been no study comparing the variability between survivors and non-survivors.

Histologic studies show the sequela of this inflammation, with severe endothelial damage, diffuse alveolar damage, thrombosis in situ, intussusceptive angiogenesis, and steroid responsive histologic patterns of organizing pneumonia (OP) and/or acute fibrosing organizing pneumonia (AFOP) [18, 19]. Dexamethasone has been shown to improve mortality in patients requiring oxygen support including invasive mechanical ventilation [20]. Our initial study suggested that low dose methylprednisolone (< 1.36 mg/kg/day) given > 7 days from onset of symptoms for 7 days were associated with improved mortality and no additional benefit with duration > 14 days or high dose (≥ 1.36 mg/kg/day) [21]. Racial/ethnic minorities have a higher incidence of chronic inflammation.

It is unknown if these groups require higher doses of methylprednisolone.

## Methodology

### Eligibility criteria

Real world data was collected from Hackensack Meridian Health (HMH), a NJ health network comprising of 13 hospitals on patients ≥ 18 years of age, and hospitalized for at least 2 days between March 1, 2020 and June 15, 2020 with severe COVID-19 Pneumonia [21]. These patients had a positive SARS-CoV-2 PCR and had SpO_2_ < 94% on room air at sea level, a respiratory rate > 30 breaths/min, PaO_2_/FiO_2_ < 300 mm Hg, or lung infiltrates > 50%. We excluded patients who had different corticosteroid regimen other than methylprednisolone. Approval was obtained by the Hackensack Meridian Health Institutional Review Board (study #Pro2020-0485) and the study was also registered on ClinicalTrials.Gov as a prospective observational database (NCT04347993).

### Data collection process and data items

Demographic data such as age, gender, race, ethnicity, comorbidities, and sex were self-reported. Weight and height were measured. SARS-CoV-2 was detected in nasal swabs by RT-PCR. Routine blood tests included complete blood count (CBC), coagulation profile, complete metabolic profile (CMP), inflammatory markers [interleukin-6 (IL-6), C reactive protein (CRP), d-dimer, and ferritin], and arterial blood gas(ABG). Data was entered into Redcap and abstracted from June to December 2020.

### Outcomes

The primary outcomes are the levels of the inflammatory markers of survivors and non-survivors and the association of methylprednisolone dose to in hospital survival for each racial/ethnic group.

### Statistical analysis

The data in this study is primarily the propensity score matched cohort of comparison of in-hospital survival of COVID-19 admitted patients that were treated with methylprednisolone and patients not treated with methylprednisolone. [21] In the one-to-one propensity score matched design of the parent study, patients from the methylprednisolone administration groups were matched based on variables associated with mortality such as age (age ≥ 60 years vs. age < 60 years), obesity (BMI ≥ 30.0 kg/m^2^ vs. BMI < 30.0 kg/m^2^), sex (M/F), diabetes (Yes/No), hypertension (Yes/No), cancer (Yes/No), respiratory Rate (respiratory rate > 22 bpm vs. ≤ 22 bpm), chronic kidney disease or chronic renal failure (Yes/No), low oxygen (oxygenation > 94% vs oxygenation ≥ 94%), CRP (CRP > 20 mg/dL vs. CRP ≤ 20 mg/dL), and qSOFA (score: 0,1,2,3) [21]. A nearest-neighbor method (greedy match) was employed using a caliper of 0.20 to obtain the propensity matched sample. A post-match assessment of the distribution of propensity scores (or logit of propensity scores) and balance in the adjusted variables between the no methylprednisolone (NMP) and methylprednisolone (MP) using standardized difference and variance ratio were performed. This analysis and graphical displays were obtained by the ASSESS statement of PROC MATCH in SAS 9.4 [21]. The aims of this subgroup analysis were pursued between and within the racial/ethnic groups that exhausted the span of racial/ethnic groups of the propensity score-matched cohort from primary study [21].

In this study descriptive statistics was used to summarize the data. Categorical variables were presented as frequencies and percentages. Continuous variables were presented as the median and interquartile range (IQR) for non-normally distributed data and mean and standard deviation for normally distributed data. The normality assumption of continuous variables was assessed using Shapiro–Wilk test. Comparison of matched continuous variables was performed using two-sided paired t-test or Wilcoxon signed-rank test, as appropriate. Comparison of paired categorical variables was performed using McNemar’s test or Bowker test of symmetry, as appropriate.

Comparison of levels of inflammatory markers, creatinine and age between racial/ethnic groups, which are independent, was performed using Kruskal–Wallis test followed by pairwise comparisons utilizing a two-sided Wilcoxon rank sum test.

Comparison of these continuous variables between survivors and non-survivors within each racial/ethnic group was performed using two-sided Wilcoxon rank sum test. Boxplots were used to illustrate the different distribution between survivor and non-survivors in the inflammatory markers. To assess the discriminative ability of inflammatory markers for in-hospital mortality, area under the receiver operating characteristics (ROC) curve of the inflammatory markers in each racial/ethnic group using ROC statements in PROC LOGISTIC SAS 9.4. Comparison of area under ROCs within each racial/ethnic group were evaluated by DeLong test, with ROC closest to 0.5 as the reference. Area under ROCs were reported as area (95% confidence interval). AUC closer to 1 with a 95% CI that excludes 1 was considered statistically significant.

Within each racial/ethnic group, this study also examined the association between in-hospital survival and inflammatory markers, methylprednisolone dose (none, low dose, high dose), older age obesity, sex, diabetes, hypertension cancer, respiratory rate (bpm), chronic kidney disease (or renal failure), supplemental oxygen support (None, non-mechanical ventilation, mechanical ventilation), CRP ≥ 20 mg/dL, qSOFA and creatinine ≥ 1.5 mg/dL. Methylprednisolone (MP) dose cutoff value of 1.36 mg/kg/day had been previously determined as optimal based on Youden index method such that low dose (LD) MP was defined as MP dose < 1.36 mg/kg/day and high dose (HD) MP was defined as MP dose ≥ 1.36 mg/kg/day. Dichotomized inflammatory marker we employed in all the time to in-hospital mortality event analysis as follows: IL-6 > 25 pg/mL, D-dimer ≥ 2 mcg/mL, CRP ≥ 20 mg/L and ferritin ≥ 1400 μg/L as high levels of each respective marker. Time to in-hospital mortality was estimated using Kaplan–Meier product limit method. Comparison of in-hospital survival between independent groups was estimated by a two-sided log-rank test and matched cohort were compared using stratified log rank. To examine the association of risk/benefit of in-hospital survival with the factor of interest such as methylprednisolone treatment, in a univariate model, the Cox proportional hazard regression analysis with robust covariance [22] (sandwich estimator) to account for paired observations was conducted. The proportional hazard (PH) assumption, critical in Cox regression modelling, was evaluated using a Kolmogorov-type supremum test [23] in the ASSESS statement of PROC PHREG. If the PH assumption was violated for any covariate, then a continuous variable which also violated the PH assumption and its interaction with time were included in the model to adjust for the significant interaction with time to the risk of mortality [24]. After conducting univariate PH Cox regression or NPH Cox regression, any covariate that reported P < 0.15 were entered into the initial full model of the multivariable Cox model to initiate backward elimination procedure. If any of covariates that satisfied the criterion of P < 0.15 above had been found to have violated PH assumption then a multivariate NPH Cox regression model was analysis, otherwise if all the variables satisfied the PH assumption then a standard multivariable PH Cox model was fit. Results of all univariate and multivariable Cox regression (PH/NPH) within each race/ethnic group were presented as hazard ratio (HR), 95% Cis and P-value. All data analysis was performed using SAS 9.4 (SAS Institute Inc., Cary, North Carolina, USA). Unless otherwise specified, any P < 0.05 was considered statistically significant.

## Results

Between March 4 and June 15, 2020, 2041 patients were flagged in the electronic health record with a diagnosis of COVID-19 and pneumonia. A total of 539 patients were excluded based on eligibility criteria (< 18 years of age, pregnant, received other formulations of corticosteroids, or hospitalized for less than 2 days) Thus, 1072 patients had their data abstracted. A propensity score matched sample was constructed out of 759 patients (380 in NMP and 379 in MP). After an examination of the proportional hazard assumption, MP and Fractional inspired oxygen (FiO_2_) significantly violated it (both with P < 0.0001). [21] Data on P/F ratio was lacking; and FiO_2_ was used since 95% of patients had this data. The supremum test also indicated that non-proportionality was observed in other variables such as nursing home, lack of taste or smell, WBC < 11,000 cells/mL, creatinine > 1.5 ng/mL, respiratory rate > 22 bpm, hydroxychloroquine (HCQ), MP, HD or LD MP, calcium, and initial diastolic blood pressure.

All variables with non-proportional hazard were adjusted using FiO_2_, as indicated above. The Youden Index method yielded a MP dose cut-off 1.36 mg/kg/day. Low dose methylprednisolone (LDMP) was defined as < 1.36 mg/kg/day and high dose methylprednisolone (HDMP) was defined as ≥ 1.36 mg/kg/day. 215 received LDMP and 164 received HDMP [21]. In the original study, there was another category for racial and ethnic group in REDCAP. We manually reviewed the charts further for racial and ethnic group and other variables, and found there were 102 Blacks, 338 Whites, 61 Asian/Indians, 251 non-white/non-black Hispanics and 7 unknown racial/ethnic group (Table 1, Additional file 1: Tables S1, S2, Figs. S1, S2).

**Table 1:** Characteristics of hospitalized COVID-19 patients treated with or without methylprednisolone (n = 759)

Level	No methylprednisolone (n = 380)	Methylprednisolone (n = 379)	P value
	Count (%)	Count (%)	
Age ≥ 60.0 years	254 (66.67)	244 (64.55)	0.2436
Male	237 (62.20)	242 (64.02)	0.5775
Black	58 (15.30)	44 (11.80)	0.2500
White	178 (46.97)	160 (42.90)	0.2500
Asian/Indian	25 (6.60)	36 (9.65)	0.2500
Hispanic	118 (31.13)	133 (35.66)	0.2500
BMI ≥ 30 kg/m^2^	179 (46.98)	181 (47.88)	0.6767
Smoker (former or current)	77 (21.69)	88 (25.96)	0.2334
Fever	250 (65.79)	294 (77.78)	0.0003
Shortness of breath	248 (65.09)	298 (79.05)	< 0.0001
Cough	242 (63.68)	270 (71.43)	0.0191
Altered mental status	63 (16.54)	41 (10.85)	0.0032
GI	76 (20.00)	81 (21.49)	0.5211
Anosmia or ageusia	6 (1.59)	9 (2.45)	0.5930
Duration of symptoms PTA	5.00 (2.00,7.00)	5.00 (3.00,7.00)	0.0699
Duration > 7 days	59 (18.91)	75 (21.99)	0.2864
Diabetes	144 (37.53)	139 (36.51)	0.6521
COPD	20 (5.25)	28 (7.41)	0.2482
Asthma	24 (6.30)	37 (9.81)	0.0741
Hypertension	225 (59.06)	219 (57.94)	0.5525
Cancer	43 (11.29)	43 (11.38)	0.9013
Cerebrovascular accident	18 (4.74)	14 (3.70)	0.3692
Coronary artery disease	29 (7.61)	28 (7.41)	0.8886
Arrhythmia	41 (10.79)	30 (7.94)	0.1213
Chronic kidney disease	28 (7.35)	31 (8.20)	0.6682
Rheumatologic disorder	10 (2.62)	19 (5.04)	0.0588
qSOFA 0	224 (58.49)	222 (58.42)	0.7647
qSOFA 1	130 (33.94)	130 (34.21)	0.7647
qSOFA 2	28 (7.31)	26 (6.84)	0.7647
qSOFA 3	1 (0.26)	2 (0.53)	0.7647
Temperature	98.80 (97.70,100.40)	99.30 (98.00,100.70)	0.1284
Heart rate	95.00 (84.00,108.00)	97.00 (86.00,108.00)	0.1438
Arterial pressure	92.33 (83.33,100.50)	90.67 (81.83,99.33)	0.0870
Respiratory rate	19.00 (18.00,22.00)	20.00 (18.00,22.00)	0.3231
O_2_ Sat < 94%	215 (56.43)	217 (57.41)	0.6733
Nasal cannula	160 (82.05)	131 (65.83)	0.2500
Venti mask	2 (1.03)	3 (1.51)	0.2500
High flow	6 (3.08)	15 (7.54)	0.2500
CPAP	1 (0.51)	2 (1.01)	0.2500
BiPAP	0 (0.00)	2 (1.01)	0.2500
Non-rebreather	26 (13.33)	46 (23.12)	0.2500
Mechanical ventilation	35 (11.15)	129 (39.33)	< 0.0001
WBC	6.60 (5.10,9.20)	6.50 (5.10,9.50)	1.0000
HGB	13.40 (12.30,14.50)	13.50 (12.20,14.70)	0.6746
PLT	203.00 (161.00,257.00)	189.50 (147.00,252.00)	0.2736
ALC	0.90 (0.60,1.30)	0.80 (0.60,1.10)	0.0031
IL6	12.00 (5.00,39.00)	15.00 (5.00,36.00)	0.2678
CRP	9.88 (4.99,17.31)	12.67 (6.84,19.08)	0.0047
D-dimer	1.09 (0.65,2.20)	1.44 (0.72,3.13)	0.1155
Ferritin	729.50 (325.50,1404.00)	867.00 (418.00,1548.00)	0.0675
Creatinine	1.01 (0.80,1.50)	1.01 (0.80,1.35)	0.2630
Troponin	0.03 (0.01,0.30)	0.02 (0.01,0.09)	0.0732
BNP	131.85 (40.30,1000.55)	88.80 (26.20,362.00)	0.2110
Hydroxychloroquine	269 (71.93)	317 (88.55)	< 0.0001
Azithromycin	255 (68.55)	264 (73.54)	0.0728
Remdesivir	3 (0.82)	10 (2.82)	0.0196
Tocilizumab	13 (3.53)	63 (17.65)	< 0.0001

**Fig. 1:**
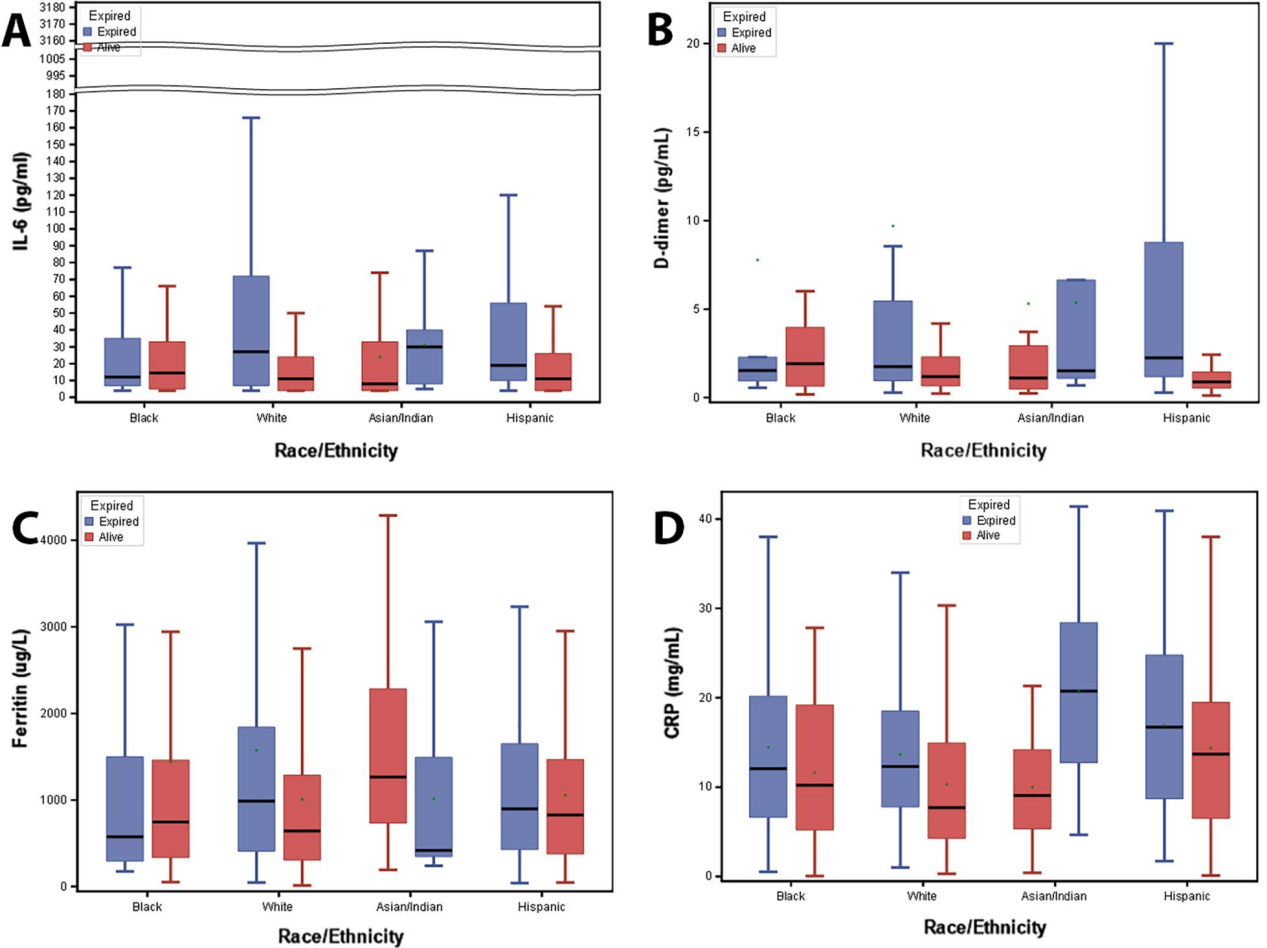
Box plots of inflammatory markers between those who expired and those who are alive. Panel shows red corresponds to those who are alive or are survivors and blue corresponds to those who have expired or are non-survivors

Box plots were created to determine the distribution of each inflammatory marker between survivors, or those who are alive, and non-survivors, or those who expired (Fig. 1). For ferritin, Asian/Indians (median = 1048 μg/L) had elevated levels compared to Blacks (median = 743.0 μg/L), Whites (median = 777.5 μg/L) and Hispanics (median = 831.0 μg/L), however, this was not statistically significant (Kruskal–Wallis P = 0.1689). Ferritin values in Blacks (n = 72) were not significantly different between survivors (n = 21) and non-survivors (n = 51) (median: 745 vs 575 μg/L, P = 0.9280). Ferritin values in Whites (n = 338) were significantly different between survivors (n = 243) and non-survivors (n = 95) (median: 641 vs 987 μg/L, P = 0.0139). Ferritin values in Asian/Indians (n = 56) were significantly different between survivors (n = 38) and non-survivors (n = 18) (median: 1265 vs 418 μg/L, P = 0.0211). The ferritin values in Hispanics (n = 226), were not significantly different between survivors (n = 158) and non-survivors (n = 68) (median: 826.5 vs 897.5 μg/L, P = 0.0854).

D-dimer values in Blacks (median = 1.64 mcg/mL), Whites (median = 1.33 mcg/mL), Asian/Indians (median = 1.21 mcg/mL) and Hispanics (median = 1.11 mcg/mL) were not significantly different (Kruskal–Wallis P = 0.1240). D-dimer values in Blacks (n = 62) were not significantly different between survivors (n = 44) and non-survivors (n = 18) (median: 1.91 mcg/mL vs 1.53 mcg/mL, P = 0.9119). D-dimer values in Whites (n = 193), were significantly different between survivors (n = 133) and non-survivors (n = 60) was significant (median: 1.19 vs 1.76 mcg/mL, P = 0.0034). D-dimer values in Asian/Indians (n = 41), were not significantly different between survivors (n = 26) and non-survivors (n = 15) (median: 1.10 vs. 1.51 mcg/mL, P = 0.0784). D-dimer values in Hispanics (n = 172), were significantly different between survivors (n = 116) and non-survivors (n = 56) (median: 0.89 vs 2.24 mcg/mL, P < 0.0001).

CRP values in Blacks (median = 11.40 mg/mL), Asian/Indians (median = 12.85 mg/mL) and Hispanics (median = 14.32 mg/mL), were statistically significantly different compared to Whites (median = 9.47 mg/mL), (Kruskal–Wallis P < 0.0001). This result was driven by the significant difference between Whites and Hispanics adjusting for multiple testing using Hochberg method. CRP values in Blacks (n = 85) were not significantly different between survivors (n = 63) and non-survivors (n = 22) (median: 10.2 mg/mL vs 12.07 mg/mL, P = 0.3586). CRP values in Whites (n = 276) were significantly different between survivors (n = 183) and non-survivors (n = 93) (median: 7.71 mg/mL vs 12.30 mg/mL, P = 0.0004). CRP values in Asian/Indians (n = 60) were significantly different between survivors (n = 38) and non-survivors (n = 22) (median: 9.05 vs. 20.75 mg/mL, P < 0.0001). CRP values in Hispanics (n = 228) were not significantly different between survivors (n = 158) and non-survivors (n = 70) (median: 13.70 mg/mL vs 16.70 mg/mL, P = 0.0514).

IL-6 values were not significantly different between the groups (Kruskal–Wallis P = 0.9843). Except for Asian/Indians with median IL-6 of 11 pg/mL, all the other groups had a median IL-6 of 14.0 pg/mL. IL-6 values in Blacks (n = 51) were not significantly different between survivors (n = 34) and non-survivors (n = 17) (median: 13 pg/mL vs 11 pg/mL, P = 0.7868). IL-6 values in Whites (n = 157) were significantly different between survivors (n = 94) and non-survivors (n = 63) (median: 11.0 pg/mL vs 27.0 pg/mL, P = 0.0005). IL-6 values in Asian/Indians (n = 61) were not statistically different between survivors (n = 38) and non-survivors (n = 23) (median 30.0 pg/mL vs 8.0 pg/mL, P = 0.0924). IL-6 values in Hispanics (n = 110) were statistically different between survivors (n = 73) and non-survivors (n = 37) (median = 19.0 pg/mL vs 11.0 pg/mL, P = 0.0168).

**Fig. 2:**
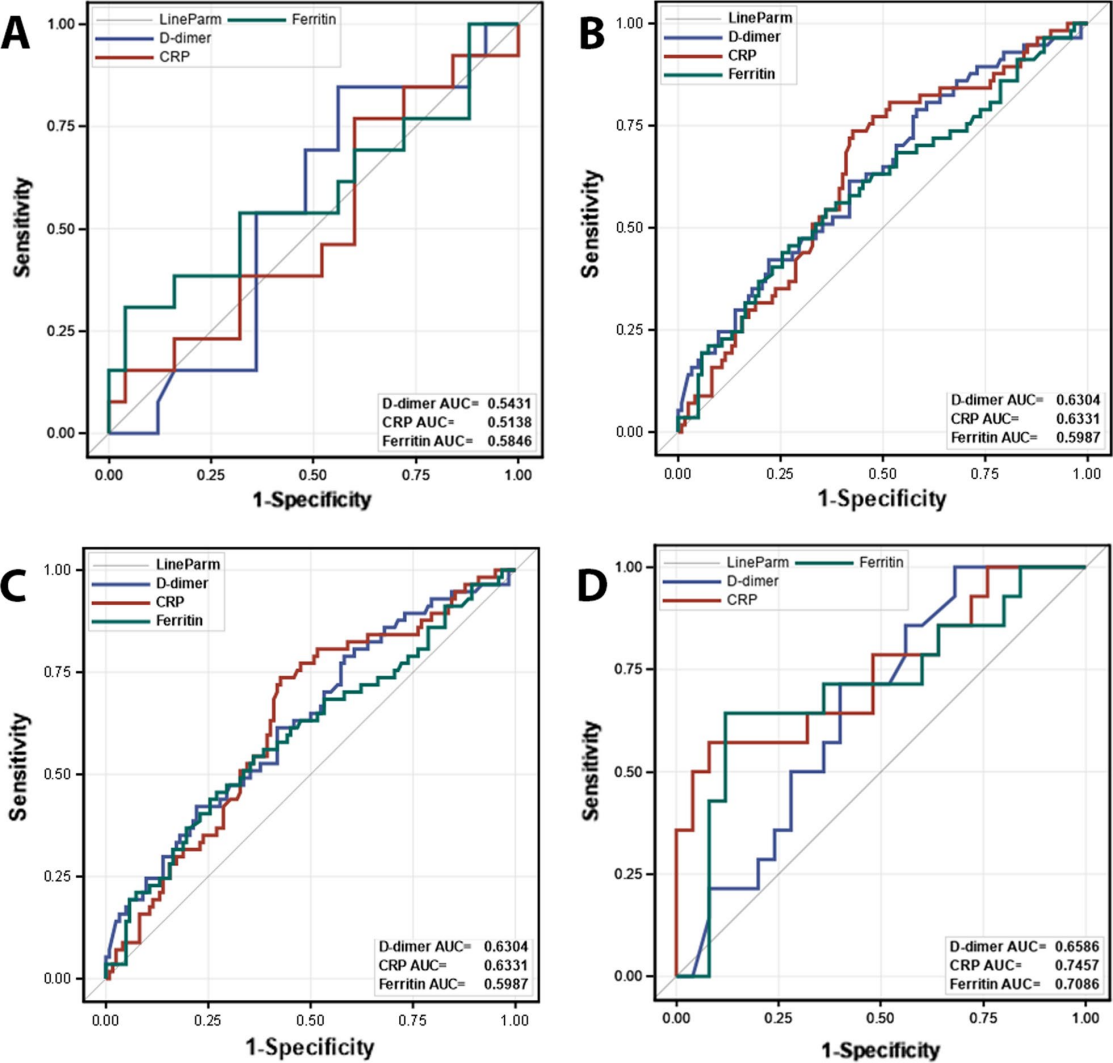
Area under receiver operator characteristic curves of inflammatory markers in each racial/ethnic group. Panel shows AUC for each inflammatory marker

ROC analysis was used to identify the candidate inflammatory markers to include in the subsequent univariate and multivariable analysis of in-hospital survival in each racial/ethnic group (Fig. 2). Since IL-6 had the most missing except for the Asian/Indian cohort, we excluded from comparative ROC analysis as the Proc LOGISTIC used to perform the AUC analysis always seeks the most common subjects between measurements in a DeLong test. Thus this analysis was limited to D-dimer, CRP and Ferritin. In the DeLong testing, we used the AUC closest to 0.5 and/or the AUC whose 95% CI included 0.5 as a reference inflammatory marker. The inflammatory marker which was significantly different from the reference by DeLong test was automatically selected as a candidate for Univariate/Multivariate Cox models. A second marker that was the second largest in the AUC value was also included regardless of lack of statistical difference from the reference marker, In cases where none of the AUCs of the markers were statistically different from the reference, we forced the markers with two largest AUCs to initiate the model fitting steps.

In Blacks, the AUCs for D-dimer (Area = 0.543, 95% CI 0.351–0.735), CRP (Area = 0.514, 95% CI 0.312–0.716), and Ferritin (Area = 0.585, 95% CI 0.372–0.797) were not significantly different, taking CRP as the reference (Delong Test P = 0.8378).

In Whites, the AUCs of D-dimer (Area = 0.630, 95% CI 0.543–0.718), CRP (Area = 0.633, 95% CI 0.547–0.719), and Ferritin (Area = 0.599, 95% CI 0.507–0.691), taking ferritin as the reference, were not statistically different (DeLong Test P = 0.8265).

In Hispanics, the AUC of D-dimer (Area = 0.742, 95% CI 0.651–0.833), CRP (Area = 0.567, 95% CI 0.472–0.662), and Ferritin (Area = 0.575, 95% CI 0.447–0.673), taking CRP as the reference, were significant (Delong Test P = 0.0038). Compared to CRP, D-dimer was statistically different (DeLong Test P = 0.0041).

In Asian/Indian, the difference between AUC of D-dimer (Area = 0.659, 95% CI 0.487–0.830), CRP (Area = 0.746, 95% CI 0.571–0.920), and Ferritin (Area = 0.709, 95% CI 0.524–0.894) is not significant (P = 0.7076). Compared to D-dimer, none of the ROC were statistically different (DeLong Test P = 0.8014).

In Blacks, univariate Cox regression revealed that increased risk of in-hospital mortality (poor in-hospital survival) was significantly associated with older age ≥ 60 years vs < 60 years (HR = 4.11, 95% CI 1.31–12.90; P = 0.0152), mechanical ventilation compared vs non-mechanical ventilation (HR = 4.36, 95% CI 1.29–14.76, P = 0.0180), qSOFA score of 2 vs 0 (HR = 2.61, 95% CI 1.16–5.89; P = 0.029), qSOFA score of 3 vs 0 (HR = 21.33, 95% CI 8.84–51.44; P < 0.0001) and creatinine ≥ 1.5 mg/dL (HR = 3.59, 95% CI 1.61–8.02, P = 0.0018) (Table 2, Additional file 1: Table S3). Univariate analysis indicated that MP dose did not violate the proportional hazard assumption NPH supremum test P = 0.7120, hence a multivariable proportional hazard Cox model was employed. As candidates in the full multivariate model, these variables along with P-values < 0.15 in the univariate analysis were entered into the subsequent multivariate’s backward elimination procedure. The extra values that met criterion for be entered in this multivariable stage included: MP dose, obesity status (BMI ≥ 30 kg/m^2^), respiratory rate > 22 bpm, and qSOFA. After the variable selection step of multivariable concluded, it was indicated that risk of poor in-hospital survival was significantly associated with older age ≥ 60 years (HR = 3.71, 95% CI 1.15–11.95, P = 0.0281), mechanical ventilation compared (HR = 5.07, 95% CI 1.42–18.07, P = 0.0123) and creatinine ≥ 1.5 mg/dL (HR = 3.61, 95% CI 1.71–7.59, P = 0.0007).

**Table 2:** Univariable and multivariate analysis of in-hospital survival in admitted COVID-19 Black patients (n = 102)

Variable/Category	Univariate analysis		Multivariate analysis	
	HR (95% CI)	P value	HR (95% CI)	P value
MP dose		0.1110		
LD MP vs NMP	0.59 (0.24,1.43)	0.2399		
HD MP vs NMP	1.48 (0.60,3.66)	0.3916		
HD MP vs LD MP	2.53 (1.06,6.06)	0.0369		
Diabetes	1.03 (0.50,2.14)	0.9348		
Hypertension	0.87 (0.36,2.10)	0.7564		
Chronic kidney disease	0.79 (0.18,3.60)	0.7653		
Male vs female	0.97 (0.47,2.01)	0.9417		
Respiratory rate > 22 bpm	1.96 (0.87,4.40)	0.1031		
Older age ≥ 60 years	4.11 (1.31,12.90)	0.0152	3.71 (1.15,11.95)	0.0281
Obesity status BMI ≥ 30 kg/m^2^	0.51 (0.23,1.11)	0.0907		
Cancer	2.28 (0.49,10.66)	0.2953		
CRP > 20 mg/L	1.45 (0.58,3.61)	0.4221		
Ferritin ≥ 1400 μg/L	1.14 (0.45,2.86)	0.7848		
Supplemental oxygen		0.0370		0.0208
NMV vs none	2.18 (0.52,9.12)	0.2839	2.63 (0.58,11.93)	0.2088
MV vs none	4.36 (1.29,14.76)	0.0180	5.07 (1.42,18.07)	0.0123
qSOFA		< 0.0001		
qSOFA 1 vs 0	1.77 (0.64,4.92)	0.2738		
qSOFA 2 vs 0	2.61 (1.16,5.89)	0.0209		
qSOFA 3 vs 0	21.33 (8.84,51.44)	< 0.0001		
Creatinine ≥ 1.5 mg/dL	3.59 (1.61,8.02)	0.0018	3.61 (1.71,7.59)	0.0007

Risk of in-hospital mortality by Cox regression model. Any P < 0.05 was statistically significant

*MV* mechanical ventilation; *NMV* non-mechanical ventilation; *HR* hazard ratio; *CI* confidence interval

In Whites, univariate Cox regression indicated that significant benefit in in-hospital survival was associated with LDMP vs NMP (HR = 0.35, 95% CI 0.22–0.56, P < 0.0001) and HDMP vs NMP (HR = 0.55, 95% CI 0.34–0.88, P = 0.0132). (Table 3, Additional file 1: Table S4) The analysis also revealed that increased risk of in-hospital mortality (poor in-hospital survival) was significantly associated with hypertension (HR = 2.03, 95% CI 1.36–3.05, P = 0.0006), respiratory rate > 22 bpm (HR = 1.66, 95% CI 1.07–2.57, P = 0.0232), chronic kidney disease (HR = 1.66, 95% CI 1.01–2.73, P = 0.0468), older age ≥ 60 years (HR = 2.22, 95% CI 1.31–3.75; P = 0.0030), cancer (HR = 1.90, 95% CI 1.24–2.92, P = 0.0032), receiving mechanical ventilation compared to no supplemental oxygen (HR = 1.72, 95% CI 1.09–2.71, P = 0.0194), qSOFA score of 1 vs 0 (HR = 2.28, 95% CI 1.53–3.38; P < 0.0001), qSOFA score of 2 vs 0 (HR = 6.83, 95% CI 4.13–11.28; P < 0.0001), qSOFA score of 3 vs 0 (HR = 8.21, 95% CI 5.64–11.95; P < 0.0001) and creatinine ≥ 1.5 mg/dL (HR = 2.02, 95% CI 1.39–2.94, P = 0.0002). In the White cohort, univariate analysis indicated that MP dose did not satisfy the proportional hazard assumption NPH supremum test P < 0.0001, hence a multivariable non-proportional hazard Cox model was employed. As candidates in the full multivariate model, these variables along with P-values < 0.15 in the univariate analysis were entered into the subsequent multivariate’s backward elimination procedure. The extra values that met criterion for be entered in this multivariable stage included sex. After the variable selection step of multivariable concluded, it was reported that significant benefit in in-hospital survival was associated with LD MP vs NMP (HR = 0.37, 95% CI 0.23–0.59, P<0.0001) and HD MP vs NMP (HR = 0.56, 95% CI 0.35–0.91, P = 0.0183). In addition, the risk of poor in-hospital survival was significantly associated with cancer (HR = 1.68, 95% CI 1.03–2.69, P = 0.0376), qSOFA score of 1 vs 0 (HR = 1.81, 95% CI 1.09–2.98, P = 0.0213), qSOFA score of 2 vs 0 (HR = 5.16, 95% CI 3.07–8.66; P < 0.0001), qSOFA score of 3 vs 0 (HR = 11.81, 95% CI 6.46–21.57; P < 0.0001) and creatinine ≥ 1.5 mg/dL (HR = 2.16, 95% CI 1.39–3.34, P = 0.0006).

**Table 3:** Univariable and multivariable analysis of in-hospital survival in admitted COVID-19 white patients (n = 338)

Variable/Category	Univariate analysis		Multivariable analysis	
	HR (95% CI)	P value	HR (95% CI)	P value
MP dose		< 0.0001		< 0.0001
LD MP vs NMP	0.35 (0.22,0.56)	< 0.0001	0.37 (0.23,0.59)	< 0.0001
HD MP vs NMP	0.55 (0.34,0.88)	0.0132	0.56 (0.35,0.91)	0.0183
HD MP vs LD MP	1.56 (0.94,2.57)	0.0855	1.53 (0.91, 2.56)	0.1076
Diabetes	1.05 (0.72,1.51)	0.8124		
Hypertension	2.03 (1.36,3.05)	0.0006		
Chronic kidney disease	1.66 (1.01,2.73)	0.0468		
Male vs female	1.38 (0.94,2.03)	0.1050		
Respiratory rate > 22 bpm	1.66 (1.07,2.57)	0.0232		
Older age ≥ 60 years	2.22 (1.31,3.75)	0.0030		
Obesity status BMI ≥ 30 kg/m^2^	1.05 (0.73,1.51)	0.8053		
Cancer	1.90 (1.24,2.92)	0.0032	1.68 (1.03,2.69)	0.0376
CRP > 20 mg/L	0.77 (0.45,1.33)	0.3500		
Ferritin ≥ 1400 μg/L	1.11 (0.74,1.67)	0.6028		
Supplemental oxygen		0.0593		
NMV vs none	1.33 (0.72,2.47)	0.3661		
MV vs none	1.72 (1.09,2.71)	0.0194		
qSOFA		< 0.0001		< 0.0001 ara>
qSOFA rec 1 vs 0	2.28 (1.53,3.38)	< 0.0001	1.81 (1.09,2.98)	0.0213
qSOFA rec 2 vs 0	6.83 (4.13,11.28)	< 0.0001	5.16 (3.07,8.66)	< 0.0001
qSOFA rec 3 vs 0	8.21 (5.64,11.95)	< 0.0001	11.81 (6.46,21.57)	< 0.0001
Creatinine ≥ 1.5 mg/dL	2.02 (1.39,2.94)	0.0002	2.16 (1.39,3.34)	0.0006

Risk of in-hospital mortality by Cox regression model. Any P < 0.05 was statistically significant

*MV* mechanical ventilation; *NMV* non mechanical ventilation; *HR* hazard ratio; *CI* confidence interval

In Hispanics, univariate Cox regression indicated that significant benefit in in-hospital survival was associated with LD MP vs NMP (HR = 0.35, 95% CI 0.18–0.67, P = 0.0015) (Table 4, Additional file 1: Table S5). In addition, the analysis indicated that increased risk of in-hospital mortality (poor in-hospital survival) was significantly associated with HD MP vs LD MP (HR = 2.08, 95% CI 1.20–3.54, P = 0.0073), hypertension (HR = 2.65, 95% CI 1.66–4.24, P < 0.0001), chronic kidney disease (HR = 2.65, 95% CI 1.38–5.09, P = 0.0034), older age ≥ 60 years (HR = 2.56, 95% CI 1.55–4.23; P = 0.0002), cancer (HR = 2.51, 95% CI 1.19–5.27, P = 0.0154), D-dimer ≥ 2 mcg/mL (HR = 1.79, 95% CI 1.05–3.04, P = 0.0320), qSOFA score of 2 vs 0 (HR = 1.99, 95% CI 1.11–3.55; P = 0.0202), and creatinine ≥ 1.5 mg/dL (HR = 2.16, 95% CI 1.20–3.89, P = 0.0100). The univariate analysis indicated that MP dose did not violate the proportional hazard assumption NPH supremum test P = 0.1120. Since creatinine > 1.5 mg/dL, which did not satisfy the proportional hazards assumption (supremum test P = 0.0190), was selected as one of the variables to initiate the multivariate model fitting process, a multivariable Non-proportional hazard Cox model was employed. As candidates in the full multivariate model, these variables were entered into the subsequent multivariate’s backward elimination procedure. No extra variables reported P < 0.15 in the univariate stage. At the conclusion of variable selection step of multivariable analysis, it was indicated that risk of poor in-hospital survival was significantly associated with hypertension (HR = 2.52, 95% CI 1.14–4.47, P = 0.0012), cancer (HR = 3.04, 95% CI 1.16–8.02, P = 0.0244) and D-dimer ≥ 2 mcg/mL (HR = 2.21, 95% CI 1.23–3.96, P = 0.0077).

**Table 4:** Univariable and multivariate analysis of in-hospital survival in admitted COVID-19 Hispanic patients (n = 251)

Variable/Category	Univariate analysis		Multivariate analysis	
	HR (95% CI)	P value	HR (95% CI)	P value
MP dose		0.0032		
LD MP vs none	0.35 (0.18,0.67)	0.0015		
HD MP vs none	0.72 (0.40,1.29)	0.2703		
HD MP vs LD MP	2.08 (1.22, 3.54)	0.0073		
Diabetes	1.20 (0.76,1.89)	0.4400		
Hypertension	2.65 (1.66,4.24)	< 0.0001	2.52 (1.14,4.47)	0.0012
Chronic kidney disease	2.65 (1.38,5.09)	0.0034		
Male vs female	1.38 (0.87,2.18)	0.1688		
Respiratory rate > 22 bpm	1.01 (0.59,1.72)	0.9774		
Older age ≥ 60 years	2.56 (1.55,4.23)	0.0002		
Obesity status BMI ≥ 30 kg/m^2^	0.75 (0.48,1.17)	0.2052		
Cancer	2.51 (1.19,5.27)	0.0154	3.04 (1.16,8.02)	0.0244
CRP > 20 mg/L	1.32 (0.84,2.08)	0.2324		
D-dimer ≥ 2 mcg/mL	1.79 (1.05,3.04)	0.0320	2.21 (1.23,3.96)	0.0077
Ferritin ≥ 1400 μg/L	1.30 (0.81,2.11)	0.2806		
Supplemental oxygen		0.2827		
NMV vs none	1.19 (0.63,2.25)	0.5961		
MV vs none	0.73 (0.35,1.53)	0.3989		
qSOFA		0.0142		
qSOFA rec 1 vs 0	0.74 (0.43,1.27)	0.2751		
qSOFA rec 2 vs 0	1.99 (1.11,3.55)	0.0202		
Creatinine ≥ 1.5 mg/dL	2.16 (1.20,3.89)	0.0100		

Risk of in-hospital mortality by Cox regression model. Any P < 0.05 was statistically significant

*MV* mechanical ventilation; *NMV* non-mechanical ventilation; *HR* hazard ratio; *CI* confidence interval

In Asian/Indians, univariate Cox regression indicated that significant benefit in in-hospital survival was associated with LDMP vs NMP (HR = 0.16, 95% CI 0.04–0.62, P = 0.0080) and HDMP vs NMP (HR = 0.24, 95% CI 0.06–0.92, P = 0.0370). (Table 5, Additional file 1: Table S6) In addition, the analysis indicated that increased risk of in-hospital mortality (poor in-hospital survival) was significantly associated with chronic kidney disease (HR = 4.77, 95% CI 1.96–11.62, P = 0.0006), older age ≥ 60 years (HR = 9.45, 95% CI 1.22–72.98; P = 0.0313), and CRP > 20 mg/L (HR = 4.46, 95% CI 1.64–12.10, P = 0.0033). In the Asian/Indian cohort, univariate analysis indicated that MP dose did not satisfy the proportional hazard assumption NPH test P = 0.0320, hence a multivariable non-proportional hazard Cox model was employed. As candidates in the full multivariate model, these variables along with variables reporting P-values < 0.15 in the univariate analysis were entered into the subsequent multivariate’s backward elimination procedure. The extra values that met criterion for being entered in this multivariable stage included: hypertension, Ferritin ≥ 1400 μg/L and creatinine ≥ 1.5 mg/dL. At the conclusion of variable selection step of multivariable analysis, it was indicated that significant benefit in in-hospital survival was associated with LD MP vs NMP (HR = 0.13, 95% CI 0.03–0.61, P = 0.0101) and HD MP vs NMP (HR = 0.15, 95% CI 0.04–0.64, P = 0.0106). In addition, risk of poor in-hospital survival was significantly associated with chronic kidney disease (HR = 6.36, 95% CI 1.87–21.67, P = 0.0031) and CRP > 20 mg/L (HR = 5.02, 95% CI 1.72–14.66, P = 0.0032).

**Table 5:** Univariable analysis of in-hospital survival in admitted COVID-19 Asian/Indian patients (n = 61)

Variable/category	Univariate analysis		Multivariate analysis	
	HR (95% CI)	P value	HR (95% CI)	P value
MP dose		0.0246		0.0237
LD MP vs NMP	0.16 (0.04,0.62)	0.0080	0.13 (0.03,0.61)	0.0101
HD MP vs NMP	0.24 (0.06,0.92)	0.0371	0.15 (0.04,0.64)	0.0106
HD MP vs LD MP	1.52 (0.45,5.12)	0.5005	1.21 (0.42,3.49)	0.7287
Diabetes	1.38 (0.54,3.54)	0.5023		
Hypertension	2.40 (0.83,6.91)	0.1047		
Chronic kidney disease	4.77 (1.96,11.62)	0.0006	6.36 (1.87,21.67)	0.0031
Male vs female	0.58 (0.23,1.45)	0.2421		
Respiratory rate > 22 bpm	1.24 (0.50,3.08)	0.6489		
Older age ≥ 60 years	9.45 (1.22,72.98)	0.0313		
Obesity status BMI ≥ 30 kg/m^2^	1.61 (0.66,3.92)	0.2922		
Cancer	1.19 (0.24,5.80)	0.8297		
CRP > 20 mg/L	4.46 (1.64,12.10)	0.0033	5.02 (1.72,14.66)	0.0032
Ferritin ≥ 1400 μg/L	0.35 (0.12,1.03)	0.0577		
Supplemental oxygen		0.5732		
NMV vs none	1.96 (0.56,6.87)	0.2914		
MV vs none	1.66 (0.38,7.26)	0.5015		
qSOFA		0.5020		
qSOFA 1 vs 0	0.90 (0.37,2.22)	0.8251		
qSOFA 2 vs 0	2.43 (0.45,13.04)	0.2987		
Creatinine ≥ 1.5 mg/dL	2.26 (0.85,6.03)	0.1031		

Risk of in-hospital mortality by Cox regression model. Any P < 0.05 was statistically significant

*MV* mechanical ventilation; *NMV* non-mechanical ventilation; *HR* hazard ratio; *CI* confidence interval

Overall, in hospital survival was significantly difference between the racial/ethnic groups (Wilcoxon P = 0.0320). This result was driven by the significant difference between Whites and Blacks (P = 0.0249) and Whites and Asian/Indians (P = 0.0463), after adjusting for multiple testing. The 30-day in hospital survival rates amongst Asia/Indians, Blacks, Hispanics, and Whites were 54.4% (95% CI 35.6–72.5%), 37.9% (95% CI 19.8–57.8%), 33.4% (95% CI 22.9–44.7%), and 41.0% (95% CI 32.6–49.6%), respectively. These differences become less significant after 30 days. There were 23 (7.42%) nosocomial infections in the no methylprednisolone group and 38 (13.72%) nosocomial infections in the methylprednisolone group (P 0.0145). The main cause of death was ARDS. (Additional file 1: Fig. S3).

Due to the varying frequencies, we were not able to compare the association of NMP, LDMP, and HDMP between racial/ethnic groups. We did compare NMP, LDMP, and HDMP and their association with survival between Whites and Hispanics and they were not significant. (P > 0.05).

## (Additional file 1: Figures S4, S5, S6) Discussion

Social determinants, through weathering and/or allostatic load theory, can lead to low-grade chronic inflammation and persistently elevated inflammatory markers at baseline [25, 26]. With lack of access to quality health care, co-morbidities such as diabetes, CKD, hypertension, and coronary artery disease can remain undiagnosed in these minority groups. This may explain why in our study some inflammatory makers were above the cut-off values for mortality even in survivors [median CRP in Black (10.2 mg/mL) and Hispanic (13.70 mg/mL)].

However, there may also be genetic influences to elevated inflammatory markers. F3 and sickle cell variant (HBB rs334) are associated with higher d-dimer levels in Blacks and thalassemia, iron overload, or high iron (HFE) mutations are associated with elevated ferritin levels in Asian/Indians [27, 28]. This can explain the elevated median d-dimer in Black survivors (1.91 pg/mL) and median Ferritin in Asian/Indian survivors (> 1265 μg/L).

In the multivariate Cox regression, D-dimer, hypertension and cancer were independently associated with higher mortality in Hispanics. D-dimer, a fragment of fibrin and marker of coagulation, has also been implicated in angiogenesis, tumor cell invasion, and metastatic spread in cancer [29, 30]. Three studies with predominantly Hispanic population on COVID-19 showed an association with d-dimer and mortality [29, 31, 32]. One study on Hispanics showed that d-dimer and hypertension were associated with worse survival [32]. A study on COVID-19 showed that d-dimer was higher in patients with hypertension compared to no hypertension [29]. Non-COVID-19 studies showed that D-dimer was associated with hypertension in Blacks [33, 34]. This was not seen in our study and maybe due to smaller sample.

In Blacks and Whites, initial creatinine ≥ 1.5 mg/dL and higher qSOFA were independently associated with higher mortality. Prehospitalization creatinine was not available in all patients to confirm a diagnosis of acute kidney injury (AKI) [35]. Mortality can reach up to 50% in hospitalized patients with COVID-19 who developed AKI and COVID-19 patients were more likely to develop AKI than those without COVID-19 [36, 37]. Causes of AKI are glomerular injury from cytopathogenic effect of SARS-COV-2 and tubular injury from right heart failure, hypovolemia, endothelial dysfunction, hypercoagulability, and mechanical ventilation settings [38–42].

CKD was independently associated with mortality in Asian/Indians. Other studies have suggested that CKD has the highest risk for mortality out of all comorbidities, and has been associated with hypercoagulopathy and elevated inflammatory markers, particularly CRP [36, 43–46].

In the univariate analysis, age ≥ 60 years was associated with worse survival in all racial/ethnic groups. However, after multivariate Cox regression, age was noted to be independently associated with mortality in Blacks only. Mechanical ventilation has been shown to have higher mortality in COVID-19 [21] and was also associated with increased mortality in Blacks only.  In this study, comorbidities may have more prognostic significance rather than age in the other racial/ethnic groups.

Chronic inflammatory states are hypothesized to cause steroid desensitization, requiring higher doses of methylprednisolone to mount an effective response. LDMP and HDMP was associated with prolonged in hospital survival in Whites and Asian/Indians. There was no additional benefit of HDMP over LDMP. Lack of benefit of HDMP maybe due to a dose dependent increased severity of critical illness polyneuropathy, increased incidence of secondary infections or practice of higher doses for patients who are very sick [47]. Methylprednisolone, regardless of dose, was not associated with improved survival in Hispanics and Blacks. This may be due to vitamin D deficiency that is common in Blacks and Hispanics [48–53]. The National Health and Nutrition Examination Survey suggested that prevalence of Vitamin D deficiency is 65.4% in Blacks, 28.9% in Hispanics, and 14% for Whites in the United States [52]. Skin pigmentation is an evolutionary adaptation to intensive solar ultraviolet radiation. Ultraviolet B (UVB) is needed to convert 7-dehydrocholesterol to Vitamin D3. The abundant melanin which contributes to brown-black pigmentation, absorbs UVR and protects from skin damage and ensures adequate Vitamin D production in low latitudes [54]. The protective benefits of abundant melanin in higher latitudes, such as in the United States, are offset by decreased production of Vitamin D [50–54]. Therefore, patients with darker skin pigmentation living in higher latitudes like United States have lower vitamin D levels compared to patients with darker skin pigmentation living in lower latitudes like Africa [54]. They would require higher amounts of ultraviolet radiation (UVR) to make the same amount of Vitamin D as those with lighter pigmentation in higher latitudes [55–60]. Vitamin D sufficiency is often associated with efficacy of steroid response [6]. This may be due to Vitamin D’s upregulation of steroid receptor GR-*α* [61] or downregulation of IL-23R driven glucorticoid resistant MDR1+ proinflammatory Th17 cells [62]. Non-steroidal anti-inflammatories such as tocilizumab maybe indicated sooner for these racial/ethnic minorities [63]. Baricitinib is promising although the studies had proportionally fewer racial/ethnic minorities [64, 65].

Strengths of this study include the median days in both methylprednisolone and no methylprednisolone were 5 days, which is at the beginning stages of inflammatory phase of the disease and there were minimal number of patients on remdesivir, which has become standard of care for COVID-19. Therefore, focus there has been on the inflammatory phase and on anti-inflammatory medications.

## Limitations

Our study has several limitations. First, since it is an observational study and there may be known and unknown confounders. However, propensity matching was employed to limit the known confounders. Second, misclassification of data is possible due to manual extraction of structured and unstructured data from medical health records. Third, there was a higher prevalence of Whites and non-white/non-black Hispanics, which might have skewed the analysis. Fourth, methylprednisolone was used as a rescue, given to patients who were at a higher risk of death. During the initial pandemic surge, there were reservations on the use of methylprednisolone due to extrapolated data on prolonging viral shedding in SARS and MERS and worse mortality in Influenza. Therefore, it was used as a rescue and reserved for patients who are already on high oxygen supplementation requirements or on mechanical ventilation.

Despite the possibility of corticosteroid resistance in certain racial/ethnic groups, the effect of corticosteroids in patients on lesser amounts of oxygen supplementation such as nasal cannula was not available.

## Conclusions

Racial and ethnic disparities in inflammatory markers preclude the use of one marker as a solitary measure of mortality. Low dose and high dose methylprednisolone were associated with prolonged survival in Whites and Asian/Indians. However, high dose was not superior to low dose to prolonging survival. Methylprednisolone, regardless of dose, was not associated with prolonged survival in Blacks and Hispanics. Large, prospective studies are needed to confirm these conclusions.

## Supplementary Information

**Additional file 1: Table S1.** Baseline demographics disease characteristics of unmatched population and propensity matched population. **Table S2.** Standardized mean differences (methylprednisolone − no methylprednisolone). **Table S3.** Non-proportional hazard assumption of covariates for In-hospital mortality in COVID-19 black patients. **Table S4.** Non-proportional hazard assumption of covariates for In-hospital mortality in COVID-19 White patients. **Table S5.** Non-proportional hazard assumption of covariates for In-hospital mortality in COVID-19 Hispanic patients. **Table S6.** Non-proportional hazard assumption of covariates for In-hospital mortality in COVID-19 Asian/Indian patients. **Figure S1.** Plot of differences methylprednisolone − no methylprednisolone in hospitalized COVID-19 patients. **Figure S2.** LPS cloud plots showing distributions of logit of propensity scores for MP and NMP treated COVID-19 patients. **Figure S3.** Kaplan Meier plot for overall in-hospital survival (IhS) for COVID-19. **Figure S4.** Kaplan Meier plot of In hospital survival of Whites versus Hispanics who received no methylprednisolone. **Figure S5.** Kaplan Meier plot of In hospital survival of Whites versus Hispanics who received low dose methylprednisolone. **Figure S6.** Kaplan Meier plot of In Hospital survival of Whites versus Hispanics who received high dose methylprednisolone.

## Data Availability

The datasets are available from the corresponding author upon reasonable request.

## Abbreviations

ABG: Arterial blood gas
AFOP: Acute fibrinous organizing pneumonia
AKI: Acute kidney injury
ARDS: Acute respiratory distress syndrome
AUROC: Area under receiver operating characteristic
BMI: Body mass index
BNP: Beta natriuretic peptide
CBC: Complete blood count
CI: Confidence interval
CMP: Complete metabolic profile
Covid-19: Coronavirus disease 2019
CRP: C-reactive protein
EHR: Electronic health record
FiO_2_: Fraction of inspired oxygen
GR-*α*: Glucocorticoid receptor alpha
HCQ: Hydroxychloroquine
HMH: Hackensack Meridian Health
HR: Hazard ratio
his: In hospital survival
IL-6: Interleukin 6
IL-23R: Interleukin 23R
IQR: Interquartile range
HDMP: High dose methylprednisolone
HR: Hazard ratio
LDMP: Low dose methylprednisolone
MDR1: Multidrug resistant mutation 1
MV: Mechanical ventilation
NMV: Non-mechanical ventilation
NMP: No methylprednisolone
OP: Organizing pneumonia
PaO_2_: Partial pressure of arterial oxygen
PH: Proportional hazard
qSOFA: Quick sequential failure assessment
RR: Relative risk
SARS-COV-2: Severe acute respiratory syndrome coronavirus 2
SpO_2_: Oxygen saturation
Th17: T helper 17 cell
UVR: Ultraviolet radiation
UVB: Ultraviolet B
