# Supplementary material for: Racial/ethnic disparities on inflammation and response to methylprednisolone in severe COVID-19 pneumonia
Source: BMC Infect Dis. 2022 Mar 14;22:254. doi: 10.1186/s12879-022-07237-1 (PMC8919360; doi:10.1186/s12879-022-07237-1)
Supplement: Supplementary file 1 — Additional file 1: Table S1. Baseline demographics disease characteristics of unmatched population and propensity matched population. Table S2. Standardized mean differences (methylprednisolone − no methylprednisolone). Table S3. Non-proportional hazard assumption of covariates for In-hospital mortality in COVID-19 black patients. Table S4. Non-proportional hazard assumption of covariates for In-hospital mortality in COVID-19 White patients. Table S5. Non-proportional hazard assumption of covariates for In-hospital mortality in COVID-19 Hispanic patients. Table S6. Non-proportional hazard assumption of covariates for In-hospital mortality in COVID-19 Asian/Indian patients. Figure S1. Plot of differences methylprednisolone − no methylprednisolone in hospitalized COVID-19 patients. Figure S2. LPS cloud plots showing distributions of logit of propensity scores for MP and NMP treated COVID-19 patients. Figure S3. Kaplan Meier plot for overall in-hospital survival (IhS) for COVID-19. Figure S4. Kaplan Meier plot of In hospital survival of Whites versus Hispanics who received no methylprednisolone. Figure S5. Kaplan Meier plot of In hospital survival of Whites versus Hispanics who received low dose methylprednisolone. Figure S6. Kaplan Meier plot of In Hospital survival of Whites versus Hispanics who received high dose methylprednisolone. [file 12879_2022_7237_MOESM1_ESM.docx]

**ADDITIONAL SECTION FOR RACIAL/ETHNIC DISPARITIES ON INFLAMMATION AND RESPONSE TO METHYLPREDNISOLONE IN SEVERE COVID-19 PNEUMONIA**

Ronaldo C. Go, Themba Nyirenda, Maryam Bojarian, Davood Karimi Hosseini, Kevin Kim, Mehek Rahim, Elli Gourna Paleoudis, Anna C. Go, Zhiyong Han, Steven J. Sperber, Anjali Gupta

**Table of Contents**

**Table S1:** Baseline Demographics Disease Characteristics of Unmatched Population and Propensity Matched Population……………………………………………….………………….………………..3

**Table S2:** Standardized Mean Differences (Methylprednisolone – No Methylprednisolone)………………………………………………..………………….…………..…..5

**Figure S1:** Plot of Differences Methylprednisolone – No Methylprednisolone in Hospitalized COVID-19 Patients……...………..…………………………………………..…………………………………6

**Figure S2:** LPS Cloud Plots showing Distributions of Logit of Propensity Scores for MP and NMP treated COVID-19 patients……………………………………………..………………………………..7

**Table S3:** Non-proportional hazard assumption of covariates for In-hospital mortality in COVID-19 black patients……………………………………………………………………………………………..8

**Table S4.** Non-proportional hazard assumption of covariates for In-hospital mortality in COVID-19 White patients……………………………………………………………………………………………9

**Table S5.** Non-proportional hazard assumption of covariates for In-hospital mortality in COVID-19 Hispanic patients…………………………………………………………………………………………10

**Table S6.** Non-proportional hazard assumption of covariates for In-hospital mortality in COVID-19 Asian/Indian patients…………………………………………………………………………………….11

**Figure S3.** KM plot for overall in-hospital survival (IhS) for COVID-19……………………………..13

**Figure S4:** Kaplan Meier Plot of In Hospital Survival of Whites versus Hispanics Who Received No Methylprednisolone…………………………………-……………………………………………….….14

**Figure S5:** Kaplan Meier Plot of In Hospital Survival of Whites versus Hispanics Who Received Low Dose Methylprednisolone…..……………………………………………………………………………15

**Figure S6:** Kaplan Meier Plot of In Hospital Survival of Whites versus Hispanics Who Received High Dose Methylprednisolone…………………………………………………….………………………….16

**Table S1.** Baseline Demographics Disease Characteristics on Unmatched Population and Propensity Score Matched Population. (22)

| **Variable** | **Unmatched No**  **methylprednisolone (N=645)** | **Unmatched Methylprednisolone (N=476)** | **P Value** | **Propensity score matched**  **No methylprednisolone**  **(N = 380)** | **Propensity Score matched Methylprednisolone (N = 379)** | **P Value** |
| --- | --- | --- | --- | --- | --- | --- |
| Age in years | 64.00(53.00,79.00) | 64.00(56.00,73.00) | 0.6620 | 65.00(54.00,80.00) | 64.00(55.00,74.00) | 0.1129 |
| Male | 411(63.43) | 219(53.03) | 0.6079 | 238(62.14) | 244 (64.21) | 0.5175 |
| Weight (kg) | 81.20(68.70,90.72) | 83.90(71.10,99.80) | 0.0040 | 81.67(70.30,95.85) | 83.90(71.45,99.80) | 0.4270 |
| BMI (kg/m^2^) | 28.18(24.83,31.65) | 29.48(25.94,34.30) | <.0001 | 29.82(25.51,32.80) | 29.69(25.82,34.31) | 0.1912 |
| White | 336(51.85) | 154(23.77) | 0.6079 | 198(51.70) | 193(52.30) | 0.5085 |
| Asian | 45(6.94) | 31(7.51) | 0.6079 | 20(5.22) | 28(7.59) | 0.5085 |
| Black | 91(14.04) | 47(11.38) | 0.6079 | 57(14.88) | 42(11.38) | 0.5085 |
| Other | 154(23.77) | 229(54.01) | 0.6079 | 94(24.54) | 96(26.02) | 0.5085 |
| Community | 527(81.33) | 250(58.96) | <.0001 | 309(80.68) | 220(7.89) | **<.0001** |
| Academic | 121(18.67) | 174(41.04) | <.0001 | 74(19.32) | 160(42.11) | **<.0001** |
| Nursing Home | 125(19.38) | 43(10.26) | <.0001 | 308(80.42) | 340(89.95) | **0.0002** |
| Former/Current  smoker | 116(19.86) | 96(25.81) | 0.0377 | 77(21.57) | 88(25.81) | 0.2334 |
| Never smoker | 468(80.14) | 276(74.19) | 0.0377 | 280(78.43) | 253(74.19) | 0.2334 |
| SOB | 412(63.78) | 334(78.96) | <.0001 | 249(65.01) | 298(78.63) | **<.0001** |
| Cough | 417(64.75) | 301(71.16) | 0.0327 | 244(63.87) | 271(71.32) | **0.0303** |
| AMS | 109(17.33) | 46(11.11) | 0.0058 | 63(16.45) | 41(10.79) | **0.0032** |
| GI | 149(23.17) | 88(20.90) | 0.4077 | 76(19.90) | 81(21.37) | 0.6545 |
| Anosmia or Ageusia | 10(1.57) | 9(2.20) | 0.4834 | 6(1.58) | 9(2.44) | 0.4445 |
| Duration of Symptoms prior to  admission | 5.00(2.00,7.00) | 5.00(3.00,7.00) | 0.0165 | 5.00(2.00,7.00) | 5.00(3.00,7.00) | 0.0523 |
| Diabetes | 214(33.18) | 157(37.20) | 0.1888 | 144(37.60) | 139(36.58) | 0.8221 |
| COPD | 27(4.19) | 30(7.09) | 0.0506 | 20(5.22) | 28(7.37) | 0.2360 |
| Asthma | 40(6.22) | 40(9.48) | 0.0569 | 24(6.27) | 37(9.76) | 0.0832 |
| Cancer | 61(9.46) | 49(11.61) | 0.2594 | 43(11.23) | 43(11.32) | 1.0000 |
| CAD | 86(13.33) | 67(15.84) | 0.2839 | 50(13.05) | 61(16.05) | 0.2594 |
| CVA | 28(4.35) | 15(3.55) | 0.6336 | 18(4.71) | 14(3.68) | 0.5887 |
| CHF | 51(7.96) | 32(7.57) | 0.9071 | 18(4.71) | 28(7.37) | 1.0000 |
| Arrhythmia | 65(10.12) | 38(8.96) | 0.5967 | 41(10.73) | 30(7.89) | 0.2126 |
| Renal Failure | 40(6.23) | 33(7.78) | 0.3249 | 28(7.31) | 31(8.16) | 0.6862 |
| Rheumatologic  Disease | 15(2.33) | 22(5.20) | 0.0159 | 10(2.61) | 19(5.01) | 0.0909 |
| qSOFA 0 | 375(59.62) | 225(54.35) | 0.2544 | 224(58.49) | 222(58.42) | 0.7647 |
| qSOFA 1 | 199(31.64) | 156(37.68) | 0.2544 | 130(33.94) | 130(34.21) | 0.7647 |
| qSOFA 2 | 51(8.11) | 31(7.49) | 0.2544 | 28(7.31) | 26(6.84) | 0.7647 |
| qSOFA 3 | 4(0.64) | 2(0.48) | 0.2544 | 1(0.26) | 2(0.53) | 0.7647 |
| O2 sat < 94% | 305(48.41) | 225(57.25) | 0.0069 | 216(56.40) | 218(57.37) | 0.8265 |
| Temperature | 99.00(98.00,100.70) | 99.30(98.00,100.80) | 0.0476 | 98.80(97.70,100.40) | 99.25(98.00,100.65) | **0.0027** |
| Heart Rate | 95.00(82.00,108.00) | 95.00(82.00,108.00) | 0.0581 | 95.00(84.00,108.00) | 97.00(86.00,108.00) | 0.2467 |
| Respiratory Rate | 19.00(18.00,21.00) | 20.00(18.00,22.00) | 0.0097 | 19.00(18.00,22.00) | 20.00(18.00,22.00) | 0.2622 |
| Nasal Cannula | 227(82.85) | 137(66.50) | 0.0005 | 161(82.14) | 132(66.00) | **0.0045** |
| Venti mask | 4(1.46) | 3(1.46) | 0.0005 | 2(1.02) | 3(1.50) | **0.0045** |
| High Flow | 8(2.92) | 15(7.28) | 0.0005 | 6(3.06) | 15(7.50) | **0.0045** |
| CPAP | 1(0.36) | 2(0.97) | 0.0005 | 1(0.51) | 2(1.00) | **0.0045** |
| BPAP | 0(0.00) | 2(0.97) | 0.0005 | 0(0.00) | 2(1.00) | **0.0045** |
| Mechanical  Ventilation | 55(10.62) | 138(39.88) | <.0001 | 35(11.08) | 129(39.09) | **<.0001** |
| WBC | 6.50(5.00,9.10) | 6.50(5.10,9.50) | 0.5947 | 6.60(5.10,9.20) | 6.50(5.10,9.55) | 0.8365 |
| HGB | 13.40(12.00,14.50) | 13.50(12.20,14.80) | 0.2190 | 13.40(12.20,14.50) | 13.50(12.20,14.70) | 0.5022 |
| PLT | 200.00(158.00,251.00) | 186.00(147.00,251.0  0) | 0.0596 | 203.00(161.00,259.0  0) | 189.50(147.00,252.0  0 | **0.0238** |

| ALC | 0.90(0.60,1.20) | 0.79(0.60,1.10) | 0.0007 | 0.90(0.60,1.30) | 0.80(0.60,1.10) | **0.0004** |
| --- | --- | --- | --- | --- | --- | --- |
| IL6 | 11.50(5.00,34.00) | 12.00(5.00,32.00) | 0.6607 | 12.50(5.00,37.50) | 12.00(5.00,31.50) | 0.8044 |
| CRP | 10.91(5.20,20.79) | 13.11(7.09,20.20) | 0.0444 | 11.71(5.34,22.10) | 13.40(7.10,20.34) | 0.3187 |
| D-Dimer | 1.01(0.64,2.11) | 0.98(0.61,1.89) | 0.7909 | 1.01(0.65,2.07) | 0.98(0.61,1.91) | 0.8118 |
| Ferritin | 641.89(320.65,1453.6  0 | 838.96(430.40,1569.  80) | 0.0044 | 727.45(331.61,1470.  50) | 853.21(444.90,1569.  80) | **0.0231** |
| Creatinine | 1.00(0.80,1.40) | 1.01(0.80,1.33) | 0.9379 | 1.01(0.80,1.49) | 1.01(0.80,1.35) | 0.2327 |
| Troponin | 0.03(0.01,0.30) | 0.02(0.01,0.09) | 0.0516 | 0.03(0.01,0.30) | 0.02(0.01,0.09) | 0.1355 |
| BNP | 103.70(29.85,701.30) | 88.80(26.20,362.00) | 0.1702 | 129.70(40.60,941.10) | 85.25(25.60,339.35) | **0.0141** |
| Hydroxychloroquin  e | 463(73.73) | 333(88.33) | <.0001 | 270(71.81) | 318(88.58) | **<.0001** |
| Azithromycin | 438(70.19) | 277(73.47) | 0.2793 | 256(68.45) | 264(73.33) | 0.1672 |
| Remdesivir | 4(0.65) | 63(16.80) | 0.0061 | 3(0.81) | 10(2.81) | 0.0512 |
| Tocilizumab | 31(5.01) | 11(2.94) | <.0001 | 14(3.78) | 64(17.88) | **<.0001** |
| Convalescent  Plasma | 0(0.00) | 4(28.57) | 0.0002 | 0(0.00) | 4(28.57) | **0.0015** |
| ECMO | 1(0.17) | 9(2.43) | 0.0011 | 1(0.28) | 9(2.54) | **0.0104** |
| Dialysis | 19(3.09) | 11(2.92) | 0.8748 | 14(3.78) | 11(3.04) | 0.6853 |

HR = Hazard Ratio; CI = Confidence Interval; SOB = Shortness of Breath; AMS = Altered Mental Status; GI = Gastrointestinal Symptoms; PTA = Prior to admission; COPD = Chronic Obstructive Disease; CAD = Coronary Artery Disease; CVA = Cerebrovascular Accident; CHF = Congestive Heart Failure; LFTs = elevated liver function tests; qSOFA = Quick Sepsis Related Organ Failure Assessment; HCQ = Hydroxychloroquine; AZ = Azithromycin; MP = Methylprednisolone; HD MP = High Dose Methylprednisolone; LD MP = Low Dose Methylprednisolone; WBC= White Blood Cells; HGB = Hemoglobin; PLT = Platelet; ALC = Absolute Lymphocyte Count; ECMO = Extracorporeal Membrane Oxygenation

**Table S2.** Standardized Mean Differences (Methylprednisolone – No Methylprednisolone. (22)

| Variable | Observations | Mean Difference | Standard Deviation | Standardized Difference | Percent Reduction | Variance Ratio |
| --- | --- | --- | --- | --- | --- | --- |
| Logit Prop Score | All | 0.18717 | 0.432253 | 0.43300 |  | 1.1296 |
|  | Region | 0.18546 |  | 0.42905 | 0.91 | 1.1395 |
|  | Matched | 0.01401 |  | 0.03242 | 92.51 | 1.0738 |
| Obesity Status  (BMI ≥ 30.0 kg/m^2^) | All | -0.12778 | 0.490334 | -0.26060 |  | 1.0820 |
|  | Region | -0.12719 |  | -0.25940 | 0.46 | 1.0812 |
|  | Matched | -0.00781 |  | -0.01593 | 93.89 | 1.0016 |
| Older Age  (>60 years) | All | -0.03502 | 0.483048 | -0.07249 |  | 0.9623 |
|  | Region | -0.03565 |  | -0.07381 | 0.00 | 0.9618 |
|  | Matched | 0.02604 |  | 0.05391 | 25.63 | 1.0353 |
| Sex | All | -0.00812 | 0.480332 | -0.01690 |  | 0.9903 |
|  | Region | -0.00871 |  | -0.01813 | 0.00 | 0.9896 |
|  | Matched | -0.02083 |  | -0.04337 | 0.00 | 0.9765 |
| Diabetes | All | -0.04801 | 0.477468 | -0.10054 |  | 1.0636 |
|  | Region | -0.04747 |  | -0.09942 | 1.12 | 1.0628 |
|  | Matched | 0.00781 |  | 0.01636 | 83.73 | 0.9914 |
| Hypertension | All | -0.05334 | 0.495965 | -0.10755 |  | 0.9754 |
|  | Region | -0.05248 |  | -0.10581 | 1.62 | 0.9756 |
|  | Matched | 0.01042 |  | 0.02100 | 80.47 | 1.0069 |
| Cancer | All | -0.02157 | 0.308368 | -0.06994 |  | 1.1960 |
|  | Region | -0.02141 |  | -0.06944 | 0.72 | 1.1942 |
|  | Matched | 0.00000 |  | 0.00000 | 100.00 | 1.0000 |
| Resp. Rate>22 bpm | All | -0.06401 | 0.396891 | -0.16127 |  | 1.2802 |
|  | Region | -0.06374 |  | -0.16059 | 0.42 | 1.2785 |
|  | Matched | -0.00781 |  | -0.01968 | 87.79 | 1.0263 |
| Renal failure | All | -0.01252 | 0.259765 | -0.04821 |  | 1.1722 |
|  | Region | -0.01241 |  | -0.04779 | 0.86 | 1.1704 |
|  | Matched | -0.00781 |  | -0.03008 | 37.62 | 1.0978 |
| Oxygen <94% | All | -0.10506 | 0.496524 | -0.21158 |  | 0.9776 |
|  | Region | -0.10428 |  | -0.21003 | 0.73 | 0.9775 |
|  | Matched | -0.01042 |  | -0.02098 | 90.08 | 0.9943 |
| CRP >20 ng/L | All | -0.06192 | 0.389826 | -0.15884 |  | 1.2908 |
|  | Region | -0.06166 |  | -0.15818 | 0.41 | 1.2891 |
|  | Matched | -0.01823 |  | -0.04676 | 70.56 | 1.0699 |
| Standard deviation of All observations used to compute standardized differences | | | | | | |

**Figure S1.** Plot of differences Methylprednisolone – No Methylprednisolone in hospitalized COVID-19 patients. The plot shows that the differences were very close to zero as were the Logit propensity scores. (22)

**Standardized Mean Differences**


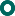

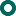

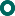

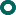

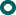

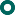

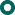

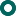

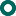


crpd1High

LowOxygen

renal_failure

respRate_high

cancer

hypertension

diabetes

sex

OlderAge

ObesityStatus

Logit Prop Score

-1.0 -0.5 0.0 0.5 1.0


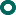

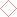
Difference (Treated - Control)

All Obs Region Obs Matched Obs Negligible differences

**Figure S2** LPS Cloud Plots showing Distributions of Logit of Propensity Scores for MP and NMP treated COVID-19 patients. The propensity matching procedure failed to match only 8 of the methylprednisolone patients with any of patients who did not receive methylprednisolone during their COVID-19 hospitalization. (22)

**LPS Clouds**

Treated


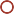

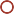

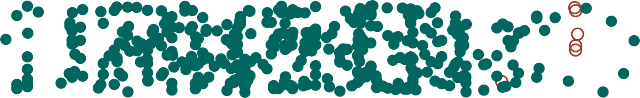

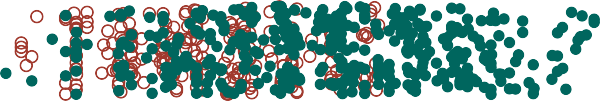


Observations

Control

-2 -1 0 1


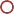
Logit of Propensity Score

Outside Support Region Support Region (Not Matched) Matched Obs

| **Table S3. Non-proportional hazard assumption of covariates for In-hospital mortality in COVID-19 black patients** | | |
| --- | --- | --- |
| Variable | Max Abs Value | P-Value |
| MP Dose | 0.5976 | 0.7120 |
| Diabetes | 0.7339 | 0.3720 |
| Hypertension | 1.0746 | 0.1760 |
| Sex | 0.8059 | 0.3110 |
| Respiratory Rate >22 bpm | 0.5980 | 0.6190 |
| Chronic Kidney Disease | 0.6506 | 0.3740 |
| Older age≥60 years | 1.0021 | 0.1500 |
| Obesity status BMI ≥30 kg/m^2^ | 0.6157 | 0.5630 |
| Cancer | 0.9525 | 0.1840 |
| CRP >20 ng/L | 0.5194 | 0.6600 |
| Ferritin ≥ 1,400 ug/L | 0.4781 | 0.7620 |
| Low oxygen | 0.5520 | 0.6940 |
| Supplemental Oxygen | 0.6788 | 0.6050 |
| qSOFA rec | 0.5417 | 0.6520 |
| Creatinine ≥ 1.5 mg/dL | 0.5623 | 0.6270 |

| **Table S4. Non-proportional hazard assumption of covariates for In-hospital mortality in COVID-19 White patients** | | | |
| --- | --- | --- | --- |
| Variable | | Max Abs Value | P-Value |
| **MP Dose** | **2.2057** | | **<.0001** |
| Diabetes | 0.5193 | | 0.7920 |
| Hypertension | 0.7370 | | 0.4230 |
| Sex | 0.8448 | | 0.2910 |
| Respiratory Rate >22 bpm | 0.8206 | | 0.3270 |
| Chronic Kidney Disease | 0.6613 | | 0.5840 |
| Older age≥60 years | 0.7540 | | 0.4080 |
| Obesity status BMI ≥30 kg/m^2^ | 0.9918 | | 0.1340 |
| Cancer | 0.8930 | | 0.2220 |
| CRP >20 ng/L | 0.6293 | | 0.5560 |
| Ferritin ≥ 1,400 ug/L | 0.6336 | | 0.5850 |
| Low oxygen | 1.0813 | | 0.1290 |
| Supplemental Oxygen | 0.7737 | | 0.3460 |
| qSOFA rec | 0.4904 | | 0.7820 |
| Creatinine ≥ 1.5 mg/dL | 1.0995 | | 0.1070 |

| **Table S5. Non-proportional hazard assumption of covariates for In-hospital mortality in COVID-19 Hispanic patients** | | |
| --- | --- | --- |
| Variable | Max Abs Value | P-Value |
| MP Dose | 1.1492 | 0.1120 |
| **Diabetes** | **1.3340** | **0.0300** |
| Hypertension | 0.9104 | 0.2130 |
| Sex | 1.0991 | 0.1220 |
| Respiratory Rate >22 bpm | 1.0615 | 0.1480 |
| Renal Failure | 0.6949 | 0.4330 |
| Older age≥60 years | 0.4140 | 0.9270 |
| Obesity status BMI ≥30 kg/m^2^ | 0.4512 | 0.9170 |
| Cancer | 0.7756 | 0.3450 |
| CRP ≥2 mcg/mL | 0.8511 | 0.3020 |
| D-dimer 2.0 | 0.5347 | 0.7910 |
| Ferritin ≥ 1,400 ug/L | 0.5895 | 0.6500 |
| Low oxygen | 0.3958 | 0.9390 |
| Supplemental Oxygen | 0.7053 | 0.5050 |
| qSOFA rec | 0.9073 | 0.3550 |
| **Creatinine ≥ 1.5 mg/dL** | **1.3665** | **0.0190** |

| **Table S6. Non-proportional hazard assumption of covariates for In-hospital mortality in COVID-19 Asian/Indian patients** | | |
| --- | --- | --- |
| Variable | Max Abs Value | P-Value |
| **MP Dose** | **1.3640** | **0.0320** |
| Diabetes | 0.3811 | 0.9100 |
| Hypertension | 0.5468 | 0.6130 |
| Sex | 0.3821 | 0.9030 |
| Respiratory Rate >22 bpm | 0.7619 | 0.4030 |
| Chronic Kidney Disease | 0.8400 | 0.2710 |
| Older age≥60 years | 0.5391 | 0.3170 |
| Obesity status BMI ≥30 kg/m^2^ | 1.1662 | 0.1000 |
| Cancer | 0.4968 | 0.6130 |
| CRP >20 ng/L | 1.0071 | 0.1300 |
| Ferritin ≥ 1,400 ug/L | 0.4932 | 0.6880 |
| Low oxygen | 0.5705 | 0.6680 |
| Supplemental Oxygen | 0.6089 | 0.5130 |
| qSOFA rec | 0.9888 | 0.2640 |
| Creatinine ≥ 1.5 mg/dL | 0.5953 | 0.5550 |

**Figure S3.** KM plot for overall in-hospital survival (IhS) for COVID-19. IhS was significantly difference between the racial/ethnic groups (Wilcoxon P=0.0320). This result was driven by the significant difference between Whites and AA (P=0.0249) and Whites and Asian/Indian (P=0.0463), after adjusting for multiple testing. The 30-day IhS rates amongst Asia/Indian, AA, Hispanics, and Whites were 54.4% (95% CI 35.6 to 72.5%), 37.9% (95% CI 19.8% to 57.8%), 33.4% (95%CI 22.9 to 44.7%), and 41.0% (95% CI 32.6 to 49.6%), respectively.


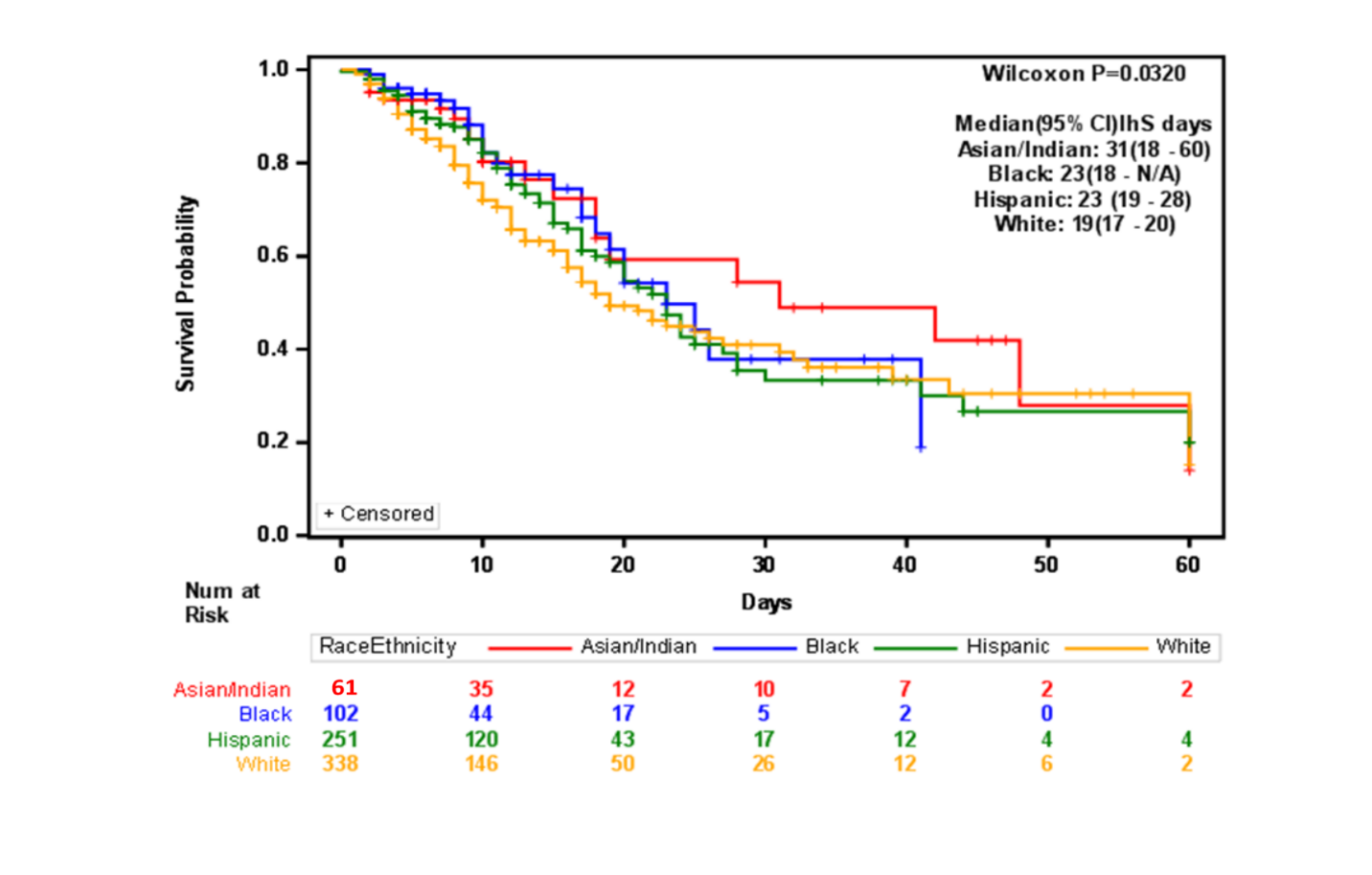


**Figure S4.** Kaplan Meier Plot of In Hospital Survival Between Whites versus Hispanics who received no methylprednisolone (NMP).

**Figure S5.** Kaplan Meier Plot of In Hospital Survival Between Whites versus Hispanics who received low dose methylprednisolone (LDMP).

**Figure S6.** Kaplan Meier Plot of In Hospital Survival Between Whites versus Hispanics who received high dose methylprednisolone (HDMP).
